# Cyber Intimate Partner Violence in Adolescents: How Do Psychopathy and Family Dynamics Shape Teens’ Online Relationships?

**DOI:** 10.3390/children12060693

**Published:** 2025-05-28

**Authors:** Alicia Tamarit, Laura Lacomba-Trejo, Francisco González-Sala

**Affiliations:** Department of Developmental and Educational Psychology, Facultat de Psicologia i Logopèdia, Universitat de València, 46010 Valencia, Spain; alicia.tamarit@uv.es (A.T.); francisco.gonzalez-sala@uv.es (F.G.-S.)

**Keywords:** adolescents, cyber intimate partner violence, psychopathy, antisocial and law-violating behaviors, child-to-parent violence, dating violence

## Abstract

Background/Objectives: Cyber intimate partner violence (CIPV) in adolescents is influenced by individual and relational factors, including psychopathic traits, antisocial and law-violating behaviors, child-to-parent violence, and dating violence. This study examines predictors of cyber aggression, cyber control perpetration, cyber victimization, and received cyber control using hierarchical regression models (HRMs) and fuzzy-set Qualitative Comparative Analysis (fsQCA). Method: A total of 207 Spanish adolescents (M = 16.18; SD = 1.52) aged 14–18 years completed measures of psychopathy (P-16), antisocial behavior (ECADA), child-to-parent violence (CTS2), and dating violence (CADRI), together with Violence in Adolescent Relationships on Social Media (e-VPA). Results: HRM showed that child-to-parent violence and experienced dating violence were common predictors across cyber aggression, cyber victimization, and received cyber control. Cyber control perpetration was mainly influenced by psychopathy and perpetrated dating violence. fsQCA revealed multiple pathways leading to high levels of CIPV, combining psychopathy, antisocial behaviors, and family and partner violence. However, cyber aggression perpetration could not be analyzed due to insufficient variability. Conclusions: The findings suggest that CIPV might stem from complex interactions between individual traits and offline relational violence. Prevention efforts should address both family dynamics and dating violence to mitigate cyber aggression and victimization in adolescent relationships.

## 1. Introduction

The ever-changing digital landscape has made technology-mediated relationships more widespread every day, especially among adolescents and young people [1]. Nowadays, online interactions are normalized in youth, facilitating contact and connection, while also encompassing risks related to online control and monitoring [2,3]. Among these risks, cyber dating violence—also referred to as cyber intimate partner violence (CIPV)—is particularly concerning, referring specifically to forms of abuse that occur within romantic or dating relationships via digital means [4]. This type of violence encompasses nuance, especially when it is studied through the paradigms of perpetration or victimization: that is, whether the person is on the enacting or receiving end of these behaviors.

Regarding perpetration within CIPV, two distinct types are cyber aggression and cyber control. Cyber aggression perpetration, on one hand, refers to hostile or threatening behaviors enacted through digital means, such as insults, humiliation, or spreading rumors [5]. Cyber control perpetration, on the other hand, involves invasive or coercive monitoring of a partner’s online activities—such as demanding access to passwords or checking messages without consent [6]. Online users may also be on the receiving end of such behaviors, reflected in cyber victimization: this includes experiencing online aggression from a partner and received cyber control, which refers to being digitally monitored, pressured, or restricted in one’s online autonomy [5,7]. As mentioned above, this specific kind of violence can vary greatly depending on the developmental stage in which it is both experienced and perpetrated, which raises concerns about its study and prevention [8].

This is particularly problematic during adolescence, a flexible developmental stage that already entails severe psychosocial and emotional changes, as it follows the person while they transition from child to adult [9]. While the World Health Organization suggests that adolescence ranges from ages 10 to 19, recent literature distinguishes between early, middle, and late adolescence, each with its unique challenges and relationship to technology [10]. This stage, overall, marked by adolescents’ heightened emphasis on peer relationships and engagement in unsupervised online activities, provides the context for the internalization and normalization of violent dating behaviors, which may then be reproduced within their own intimate relationships [5,7,11]. In adolescence, CIPV is an increasingly debated, researched, and worrisome topic since it is accompanied by the enormous social and technological changes that define the current era and affect young people the most [3,12]. Mirroring patterns of offline abuse within the digital sphere, CIPV engages in aggression, control, harassment, and surveillance within romantic relationships in online contexts, providing enhanced feelings of immunity towards the perpetrator and vulnerability in the victim [7,13]. This phenomenon has a strong gender factor: literature on cyber dating violence has observed a close connection between sexting (sending or receiving messages of sexual nature) and CIPV, and this relationship is stronger for boys than for girls during adolescence [14]. Additionally, girls appear to be more likely to face negative consequences of CIPV, and boys have been observed to be more engaged in control or harassment behaviors over girls in online settings, suggesting that gender inequality could be a driver of this kind of violence in adolescence [4]. Although research has observed girls to experience cyber victimization at a higher rate than boys, it is often associated with online contact with strangers [15]; thus, gender roles in CIPV remain a debate in scientific literature [16]. From this perspective, what other individual traits could be playing a role in the perpetration or experience of cyber intimate partner violence?

The literature indicates that individual traits can be associated with being the one responsible for or victim of certain kinds of violence, especially those associated with empathy and aggressiveness, such as psychopathic traits, antisocial and law-violating behavior, and child-to-parent violence (CTPV) [17,18,19]. Psychopathic traits, particularly when related to callousness, impulsivity, and interpersonal manipulation, have been linked to reduced empathy and increased likelihood of engaging in manipulative or aggressive behaviors in intimate contexts like romantic relationships [19,20]. Interestingly, psychopathic traits do not only associate with cyber dating violence perpetration but also with victimization—having experienced this kind of cyber violence in the past can also be associated with Dark Tetrad traits, which include subclinical narcissism, Machiavellianism, subclinical psychopathy, and everyday sadism [16]. These traits may contribute to the normalization of controlling or harmful behaviors within adolescent relationships, both online and offline. In parallel but stemming from the study of the underlying traits of externalizing behavior, antisocial and law-violating behavior has similarly been identified as a risk factor for CIPV [21]. The literature on this factor, which includes a broad spectrum of norm-violating actions such as aggression or rule-breaking, suggests that adolescents who display antisocial tendencies may be more likely to engage in digital forms of harassment, threats, or coercive control [19]. These behavioral expressions of externalizing traits often intersect with other psychologically rooted patterns of violence, such as child-to-parent violence—this factor, which refers to physical, psychological, or financial abuse directed at parents or caregivers, has been observed to work as a predictor of patterns of family dysfunction and aggression [22]. While it is often classified behaviorally, CTPV is increasingly understood as a psychologically rooted pattern of violence that is associated with deficits in emotional regulation, poor impulse control, and maladaptive relational schemas [23]. There is a consensus in the literature suggesting that child-to-parent violence presents higher rates when directed towards the mother than the father, especially when perpetrated by boys [24]. In any case, adolescents who engage in these types of behaviors often show difficulties in emotional regulation and conflict resolution, which may also appear in peer and romantic relationships, increasing the likelihood of their involvement in CIV as either perpetrators or victims [25,26]. Furthermore, and in line with these violence-related traits and behaviors, the literature supports a traditional belief in psychology studies: past behavior predicts future behavior [27,28]. Therefore, previous experiences of dating violence, online or offline, have been found to strongly predict CIVP in adolescents [29]. These individual risk factors often interact with intimate relational dynamics to shape adolescents’ experiences and behaviors within their digital romantic interactions [4,30]. However, these individual traits do not exist in a vacuum—social and cultural contexts play a significant role in the way adolescents establish patterns of communication, regulate their emotions, or engage in violent behaviors [31].

In that sense, Spanish adolescents have been observed to be at a particular risk of CIPV, given their extensive use of social media—it is estimated that they spend over an hour daily on social media apps such as TikTok and Instagram, making these part of their everyday lives [32]. Online spaces associated with violence—especially those linked to gender, such as dating violence—are increasingly becoming a part of mainstream discourse and daily use, fueling aggression and control in intimate relationships among Spanish adolescents [33]. Studies addressing the prevalence of CIPV in Spanish youth are scarce, although they point to alarming ranges: CIPV seems to be perpetrated by boys in a range from 9.70% to 44.10%, and 13.50% to 55.10% for girls, while it is disclosed to be experienced by 46% to 58.30% of boys and 57% to 60.10% of girls, depending on the study [34,35]. Despite this urgency to address violence in online settings among youth in Spain, few studies have been conducted on this topic that combine the individual risk factors mentioned above [11]. Moreover, most of them have been conducted through linear methodologies such as hierarchical regression models (HRMs), likely missing key information about how these variables interact in the prediction of CIPV. Thus, the use of non-linear methodologies such as fuzzy-set Qualitative Comparative Analysis (fsQCA) in combination with traditional linear analyses could provide a more comprehensive understanding of the complex and configurational nature of CIPV, identifying multiple pathways and combinations of factors that lead to similar outcomes.

To this purpose, the current study aims to analyze the impact of psychopathic traits, antisocial behavior, child-to-parent violence, and dating violence on cyber intimate partner violence through a mixed-methods design. We hypothesize that (1) psychopathic traits, antisocial behaviors, child-to-parent violence, and dating violence will be positively associated with CIPV; (2) cyber aggression and cyber control perpetration will be predicted by psychopathic traits, antisocial behaviors, child-to-parent violence, and dating violence; and (3) CIPV will be linked to high levels of psychopathy, high levels of antisocial behaviors, high child-to-parent violence towards both the mother and the father, and high levels of perpetrated or experienced dating violence.

## 2. Materials and Methods

### 2.1. Participants

This study included a sample of 207 Spanish adolescents between the ages of 14 and 18, with a mean age of 16.18 years (*SD* = 1.52). Most participants were female (65.90%), and 94.20% held Spanish nationality. Most students (55.60%) were enrolled in Compulsory Secondary Education (ESO), while 27.40% were in Bachillerato, and the remaining 17.00% were attending university. Informed consent was obtained from both the participants and their parents.

### 2.2. Variables and Instruments

Sociodemographic variables, including gender, age, nationality, and academic level, were collected using a custom-designed measure. Additionally, various validated questionnaires proven to have strong psychometric properties in similar populations were used to assess key variables. The selection of these instruments was carefully made to ensure a thorough and reliable evaluation.

- Violence in Adolescent Relationships on Social Media (e-VPA): This instrument consists of 20 items divided into four subscales assessing violent behaviors towards a partner and experiences of victimization through social networks. It includes two main subscales, each containing 10 items: e-victimization and e-violence. Additionally, these subscales provide specific scores for cyber violence perpetrated, cyber control perpetrated, cyber victimization, and cyber control received. Previous studies have demonstrated good internal consistency and an adequate factorial structure [6].
- Psychopathy was assessed using the Psychopathy Content Scale (P-16), originally developed by Salekin et al. [36]. This scale was derived from the Millon Adolescent Clinical Inventory (MACI), a 160-item self-report measure designed to evaluate personality traits and psychopathology in adolescents. The P-16 was constructed by selecting items from the MACI that theoretically aligned with the Hare Psychopathy Checklist-Revised (PCL-R) and corresponded to the psychopathy models proposed by Cooke and Michie [37] and Frick et al. [19].
- The final version of the P-16 consists of 16 dichotomous items (True/False), which are categorized into three subscales: callousness, egocentricity, and antisociality. The total score, obtained by summing the subscale scores, was used in this study. In the original validation, the internal consistency for the total scale was α = 0.86, while the reliability values for the subscales were 0.62 (callousness), 0.61 (egocentricity), and 0.56 (antisociality) [36].
- Antisocial and law-violating behaviors: the Antisocial and Criminal Behavior Scale in Adolescents (ECADA) was used to assess antisocial and law-violating behaviors [38]. This scale consists of 25 True/False items that measure different forms of delinquent conduct. The items are distributed across five key dimensions: early antisocial tendencies, vandalism, property-related offenses, violent acts, and substance use (alcohol and drugs). For this study, a total score was computed by summing all subscales, with higher scores reflecting greater engagement in antisocial and law-violating behaviors. The ECADA has demonstrated strong psychometric properties, with internal consistency reliability coefficients ranging from α = 0.82 to 0.86 [38,39].
- Child-to-parent violence was evaluated using the Conflict Tactics Scales (CTS2)—children-to-parents version [40,41]. For this study, the adaptation developed by the Lisis Group [42] was applied. The instrument consists of 10 items answered separately for mother and father using a five-point Likert scale ranging from 0 (Never) to 4 (Many times). The scale provides both a global index of child-to-parent violence and scores for three distinct dimensions: verbal violence, physical violence, and economic violence. In this study, the total score was obtained by summing the subscale scores. This version of the scale has demonstrated satisfactory psychometric properties, with internal consistency indices ranging from α = 0.66 to 0.85 across subscales [42].
- Dating violence was assessed using a brief version of the Conflict in Adolescent Dating Relationships Inventory (CADRI) [43,44]. This study employed the adaptation by the Lisis Group [42], which consists of 34 items evenly divided between violence perpetrated (17 items) and violence received (17 items). The items are categorized into three dimensions: relational violence, verbal-emotional violence, and physical violence. Responses are recorded on a four-point scale, ranging from 0 (Never) to 3 (Frequently; six or more times). In this study, only received physical violence and received verbal-emotional violence were analyzed to assess dating violence. The original version of the scale reported an internal consistency of α = 0.83 [44], while the Spanish adaptation obtained α = 0.86 [43].

### 2.3. Procedure and Design

This study employed a convenience sampling method, selecting adolescents from various educational institutions in the Valencian Community based on their availability and willingness to participate. The sample included individuals aged 14 to 18 years, ensuring diversity while maintaining a manageable group size.

Before data collection, researchers identified appropriate assessment instruments and contacted educational centers through an introductory letter explaining the study. Follow-up communication was conducted via phone and email to discuss participation with school administrators. Upon obtaining their approval, further meetings were arranged to provide detailed information about the research. Necessary authorizations, including informed consent from the Regional Ministry of Education and parental permissions, were secured to allow students to complete the surveys.

The surveys were administered in a single one-hour session, after which responses were systematically recorded in a database for later statistical analysis. The results were then processed and interpreted to draw relevant conclusions.

To ensure methodological rigor, a pilot study was conducted with a group of university students, who assessed the clarity of the questions and estimated completion time. This process allowed researchers to analyze the quality of the application, ensuring the adequacy of the questions and the allocated time, and they suggested no further changes. This study had a cross-sectional design.

The study followed ethical research standards, complying with the principles outlined in the Helsinki Declaration, including participant rights to informed consent, privacy, data confidentiality, non-discrimination, voluntary participation, and the option to withdraw at any point. Ethical approval was obtained from the Ethics Committee of the Universitat de València (REF 2024-PSILOG-3592610).

### 2.4. Analysis

Descriptive analyses were conducted to characterize the sample and determine calibration values. Additionally, Pearson correlations were performed. To address the study’s objectives, we applied two complementary analytical strategies: hierarchical regression analysis and fuzzy-set Qualitative Comparative Analysis (fsQCA). This mixed-methods approach allowed us to examine both the net effects of individual predictors and the complex configurational patterns that may lead to cyber intimate partner violence. Importantly, the integration of both methods serves distinct but complementary purposes in testing our hypotheses. While hierarchical regression evaluates the individual and additive effects of predictors, fsQCA identifies the synergistic and conditional combinations of factors that lead to the same behavioral outcome. The analyses were carried out using SPSS v28 (IBM Corp., Armonk, NY, USA) and fsQCA 3.2 (University of California, Irvine, CA, USA).

Hierarchical regression analyses were conducted separately for four outcome variables: perpetrated cyber intimate partner aggression, perpetrated cyber intimate partner control, experienced cyber intimate partner aggression, and experienced cyber intimate partner control. A three-step hierarchical approach was employed to assess the incremental contribution of theoretically grounded predictors. In Step 1, the model included two individual-level psychological traits: psychopathy and antisocial behavior, both of which have been linked to externalizing and aggressive behaviors in previous literature. In Step 2, we added family-related contextual variables, specifically exposure to child-to-parent violence directed at the mother and father, as proxies for learned patterns of interpersonal aggression. In Step 3, the models incorporated prior experiences with offline dating violence, both as perpetrator and victim, in order to examine whether engagement in offline violent dynamics contributes to the perpetration or experience of cyber intimate partner violence.

Fuzzy-set Qualitative Comparative Analysis is a configurational analytical approach that explores how different conditions work together to bring about a particular outcome [45]. In contrast to conventional regression techniques—which typically assume linear, additive, and independent effects—fsQCA allows for the identification of multiple, equally valid combinations of variables (equifinality) that can lead to the same result. This makes it especially valuable in social science research, where variable relationships are often complex, non-linear, and influenced by context. Regarding this analysis, missing data were first removed, and recalibration was performed based on three threshold settings: 0% (low level/exclusion), 50% (intermediate level/partial membership), and 90% (high level/full inclusion). Next, the necessary and sufficient conditions were calculated. Necessary conditions are those that must always be present for an outcome to occur, while sufficient conditions can lead to an outcome but are not always required.

QCA models evaluate explained variance, measure the proportion of cases where the model applies (coverage), and assess model accuracy (consistency). A condition is considered necessary if its consistency is ≥0.90, while a model is deemed sufficiently informative if its consistency is around or above 0.74. In the fsQCA analysis, both the intermediate and complex solutions were combined to account for core and peripheral conditions, ensuring a more comprehensive interpretation of the configurational pathways leading to cyber intimate partner violence. Unlike conventional regression techniques, this approach emphasizes the combinations of conditions that lead to specific outcomes rather than isolating the independent effect of each variable.

Additionally, it was not possible to calculate the cyber aggression perpetration model using QCA due to the lack of variability in this variable. The homogeneity in responses prevented the identification of sufficient diversity in causal conditions, making it unfeasible to extract meaningful configurational patterns for cyber aggression. However, the other subfactors of cyber control perpetrated, cyber victimization, and received cyber control were analyzed as planned.

## 3. Results

### 3.1. Descriptive Statistics and Mean Comparisons

Mean comparison analyses were conducted to examine whether there were significant gender differences in the study variables. For this purpose, independent sample *t*-tests were calculated for each variable.

As shown in Table 1, statistically significant differences were found between boys and girls in the following variables: psychopathy (*t*_199_ = 2.02, *p* = 0.020, *d* = 0.30) and antisocial behavior (*t*_138_ = 3.28, *p* = 0.001, *d* = 0.72). In both cases, men scored higher than women. No statistically significant gender differences were observed for the remaining variables.

### 3.2. Correlational Analysis

Moderate, positive, and statistically significant linear associations were found among all the studied variables. However, child-to-parent violence towards the father showed no significant relationship with cyber violence in romantic relationships, nor did psychopathy correlate with cyber violence victimization in relationships. Overall, different forms of violence (directed towards parents and romantic partners), psychopathy, and delinquent behaviors were interrelated. More information can be found in Table 2.

### 3.3. Predictive Analysis

#### 3.3.1. Hierarchical Regression Models

The study aimed to predict cyber dating aggression (including both cyber aggression and cyber control) and cyber victimization (cyber victimization and received cyber control) using a hierarchical model. The predictors were introduced in three steps: antisocial and law-violating behaviors and psychopathy in the first step, child-to-parent violence (towards both mother and father) in the second step, and intimate partner violence (both perpetrated and experienced) in the third step.

The prediction model for cyber aggression accounted for 23% of the variance, with child-to-parent violence towards both parents and experienced intimate partner violence emerging as significant predictors. Meanwhile, the cyber control perpetration model explained 40% of the variance, primarily influenced by psychopathy and perpetrated intimate partner violence.

Regarding cyber victimization, the model explained 34% of the variance in the final step, with child-to-parent violence towards both parents and experienced intimate partner violence as key contributors. Lastly, the prediction model for received cyber control accounted for 36% of the variance, with child-to-parent violence towards the father and experienced intimate partner violence as significant predictors. Table 3 presents the detailed results of the hierarchical regression models.

#### 3.3.2. Fuzzy-Set Qualitative Comparative Analysis

##### Analysis of Necessary Conditions

First, the primary descriptive statistics and calibration values for the study variables are presented (Table 4). The analysis revealed that none of the examined conditions met the threshold for being considered necessary for predicting high or low levels of cyber dating aggression (including both cyber aggression and cyber control) or cyber victimization (cyber victimization and received cyber control). This conclusion was made since the consistency values remained below 0.90 in all cases (Table 5), as recommended by Ragin (2008) [45].

##### Analysis of Sufficiency Conditions

In the sufficiency analysis, the combinations of conditions that led to high and low levels of cyber control perpetrated, cyber victimization, and received cyber control were identified (Table 6). Following the criteria established in fsQCA, where a model is considered informative when consistency values are around or above 0.74 [46], all the models met this threshold, ensuring robust findings.

For high levels of perpetrated cyber control towards the partner, five pathways were identified, collectively explaining 72% of the cases (overall consistency = 0.77; overall coverage = 0.72). The most significant configuration involved the presence of psychopathy, perpetrated intimate partner violence, and experienced intimate partner violence, accounting for 62% of the cases. Another relevant pathway, explaining 54% of the cases, was characterized by experienced and perpetrated intimate partner violence combined with child-to-parent violence towards the mother. A third configuration, which explained 35% of the cases, included psychopathy, antisocial and law-violating behaviors, experienced intimate partner violence, and the absence of child-to-parent violence towards the father.

For low levels of perpetrated cyber control towards the partner, five pathways were identified, explaining 87% of the cases (overall consistency = 0.77; overall coverage = 0.87). The most relevant configuration considered solely the absence of experienced intimate partner violence, accounting for 75% of the cases. Another key pathway, explaining 73% of the cases, was defined by the absence of perpetrated intimate partner violence. Additionally, a third pathway, explaining 51% of the cases, involved the absence of psychopathy and antisocial and law-violating behaviors.

For cyber victimization, a total of 13 pathways were identified, collectively explaining 96% of the variance (overall consistency = 0.74; overall coverage = 0.96). Among these, one of the most relevant configurations, explaining 46% of the cases, was defined by the absence of psychopathy and child-to-parent violence towards both parents. Another configuration, which also accounted for 46% of the variance, combined the presence of psychopathy, antisocial and law-violating behaviors, and both perpetrated and experienced intimate partner violence. Additionally, another meaningful pattern, explaining 44% of the cases, was characterized by the absence of delinquent behaviors, absence of child-to-parent violence towards both parents, and absence of experienced intimate partner violence.

The analysis of low levels of cyber victimization revealed eight pathways, which together accounted for 77% of the variance (overall consistency = 0.87; overall coverage = 0.77). A prominent configuration, explaining 49% of the cases, was defined by the presence of perpetrated intimate partner violence and the absence of experienced intimate partner violence. Another relevant combination, explaining 38% of the cases, included the absence of antisocial and law-violating behaviors, the absence of experienced intimate partner violence, and the presence of child-to-parent violence towards the father. A further pattern, explaining 36% of the cases, was characterized by the presence of psychopathy, the absence of antisocial and law-violating behaviors, the presence of child-to-parent violence towards the mother, and the presence of experienced intimate partner violence.

Regarding the experienced cyber control, three pathways explained 51% of the variance (overall consistency = 0.81; overall coverage = 0.51). The first configuration, which accounted for 35% of the cases, was characterized by the presence of psychopathy, antisocial and law-violating behaviors, perpetrated and experienced intimate partner violence, and the absence of child-to-parent violence towards the father. A second pathway, explaining 33% of the cases, included a similar combination but with the absence of child-to-parent violence towards the mother. The third configuration, explaining 31% of the cases, involved child-to-parent violence towards both the mother and the father, perpetrated and experienced intimate partner violence, and the absence of antisocial and law-violating behaviors.

For low levels of experienced cyber control, seven pathways were identified, collectively explaining 72% of the cases (overall consistency = 0.88; overall coverage = 0.72). The most relevant configuration was characterized by the absence of antisocial and law-violating behaviors, absence of perpetrated intimate partner violence, and absence of experienced intimate partner violence. Another key pathway, accounting for 46% of the cases, involved the absence of antisocial and law-violating behaviors, absence of child-to-parent violence towards the mother, and absence of experienced intimate partner violence. Additionally, a third relevant pattern, explaining 44% of the cases, was defined by the absence of child-to-parent violence towards both the mother and the father, as well as the absence of both perpetrated and experienced intimate partner violence.

## 4. Discussion

The present study aimed to analyze the impact of psychopathic traits, antisocial behavior, child-to-parent violence, and dating violence on cyber intimate partner violence through a mixed-methods approach. It specifically examined cyber violent behaviors within the context of social media platforms, analyzing them independently from other digital contexts (e.g., messaging apps, online gaming, or anonymous forums). This research presents a strong contribution to research by filling the gap in the literature on CIPV and its predictors, analyzing them in combination, and finding the specific configurations in which they jointly operate. Psychopathic traits, antisocial behavior, child-to-parent violence, and dating violence have unique relationships with CIPV while also interacting in distinct ways that create multiple pathways to both perpetration and victimization, highlighting the multifactorial nature of adolescent cyber intimate partner violence. Furthermore, this study provides a novel contribution by combining hierarchical regression models and fuzzy-set Qualitative Comparative Analysis to explore predictors of CIPV among adolescents, offering both linear and configurational perspectives.

Our first hypothesis stated that psychopathic traits, antisocial behaviors, child-to-parent violence, and dating violence would be positively associated with CIPV. Correlation and HRM analyses partially support this hypothesis. Psychopathic traits were related to all forms of CIPV except cyber violence victimization in relationships. This aligns with previous research indicating that there exists a relationship between psychopathic traits and all dimensions of CIPV, both linked to perpetration and experience of dating online violence [16,20]. Similarly, antisocial and law-violating behaviors have also been found to correlate with all kinds of CIPV, aligning with previous research on the topic [21]. These associations might be due to the normalization of aggression and violence in Spanish adolescents, in which lower levels of empathy could play a significant role in facilitating both perpetration and experience of CIPV. This interpretation stems from previous research indicating that lower levels of empathy are associated not only with the perpetration of dating violence but also with greater acceptance and tolerance of victimization in adolescent relationships, which could indicate the presence of underlying traits such as psychopathy rather than merely social or cultural factors [48,49]. Regarding child-to-parent violence, it presented a significant relationship with CIPV when directed towards the mother but not directed towards the father. This result is consistent with previous literature indicating that there is a significant difference between the violence perpetrated towards mothers of fathers, the former being more frequent and probably linked to other kinds of violence, such as CIPV [24]. Additionally, both perpetrated and experienced intimate partner violence were positively associated with all dimensions of CIPV, suggesting that cyber violence in these settings might mirror offline forms of intimate partner violence for adolescents, as previous literature also observed [29]. These findings support the idea that CIPV is embedded within a broader pattern of aggression and transgressive behaviors in adolescence, though not all variables equally contribute across all forms of cyber violence.

As for gender differences, these findings align only partially with previous literature highlighting gender as a central factor in cyber intimate partner violence during adolescence. While earlier studies suggest that boys are more likely to engage in controlling or harassing behaviors online and that girls are more often exposed to their negative consequences [4,14], the present results did not reveal significant gender differences in the perpetration or experience of CIPV. However, boys in the sample did report higher levels of psychopathy and antisocial behavior compared to girls, which may point to a greater underlying tendency toward aggressive or transgressive behavior among male adolescents. This suggests that, rather than gender alone, certain individual traits could be key contributors to the dynamics of cyber violence in adolescent relationships. These findings add nuance to the existing debate on the role of gender in CIPV [16] and underscore the importance of considering dispositional variables when examining digital aggression in youth.

Our second hypothesis proposed that psychopathic traits, antisocial behavior, child-to-parent violence, and dating violence would predict cyber aggression and cyber control perpetration. Our results partially support this hypothesis, expanding on the correlations with all dimensions of CIPV previously found. On the first step, the model including psychopathy and antisocial behavior was significant overall, with antisocial behavior being a significant predictor on all dimensions of CIPV, and psychopathy only being significant to forms of cyber control but not aggression, both perpetrated and experienced. The relationship between psychopathy and the different forms of intimate partner violence has been addressed in literature, but the topic is still debated—contrary to previous research, psychopathy seems to be more associated with manipulative and controlling forms of violence rather than direct aggression in Spanish adolescents [16].

In the second step, adding child-to-parent violence added a significant amount of variance in perpetrated and experienced forms of violence but not control, with violence towards the mother being a positive predictor and towards the father, negative. This might indicate that Spanish adolescents who engage in aggressive forms of CIPV tend to be more violent towards their mothers and have more respect for their fathers. Previous research has addressed these distinct adolescents’ gender perceptions of their caregivers: traditional gender roles are strongly linked with sexism in domestic settings, in which mothers become the main caregivers and attachment figures, and fathers adopt a more detached position [24]. This, together with the increasing discourse of male supremacy and anti-feminism found in online settings (as well as in family dynamics), could lead adolescents who are familiarized with CIPV to be violent towards their mothers while respecting, and even idolizing, their fathers [4]. This suggests a potential line of research exploring whether the fathers’ attitudes towards their female spouses could mediate these gender-based dynamics.

The final model, tested in the third step, provided the highest amount of explained variance by adding perpetrated and experienced dating violence as predictors. As observed in previous literature, perpetrated violence in intimate settings was a predictor of perpetrated CIPV, and experienced violence strongly predicted experienced CIPV, the latter being the strongest predictor [29]. These results suggest that adolescents who have been victims of offline dating violence may be more vulnerable to being involved in online forms of violence, either as a result of trauma, normalization of controlling behaviors, or reciprocation in the digital space. This highlights the importance of understanding CIPV not in isolation but as part of a continuum of violence that spans offline and online interactions.

The third and last hypothesis posited that CIPV would be linked to high levels of psychopathy, high levels of antisocial behaviors, high child-to-parent violence towards both the mother and the father, and high levels of perpetrated or experienced dating violence, as indicated by previous research. Our results partially support this hypothesis: while HRM provided useful information about the linear relationships between CIPV and its predictors, fsQCA analyses shed light on non-linear associations, adding important nuance and explaining these relationships in a different, richer paradigm.

Starting with perpetrated cyber control, the strongest predictor across pathways was the presence of experienced intimate partner violence, closely followed by perpetrated intimate partner violence, supporting previous research on the topic [7,13]. These results suggest that engaging in controlling forms of violence towards partners is linked to a profile of adolescents who both engage in and endure violence in their relationships. The presence of psychopathic traits also emerged as a strong predictor in two of the three main pathways for this type of violence [16]. Together with the previous predictors, this suggests a reciprocal and instrumental dynamic of intimate partner violence, where control is used deliberately, perhaps to preempt or retaliate against perceived threats. Furthermore, being the perpetrator of CIPV was linked to profiles of both the presence of child-to-parent violence towards mothers and the absence of this type of violence towards fathers [25,50]. This further supports our previous hypothesis that Spanish adolescents who are violent towards their romantic partners might have a profile rooted in sexist ideas of men and women, facilitating contempt towards their mothers and respect towards their fathers. This interpretation is consistent with research suggesting that adolescents who endorse traditional or sexist gender norms may reproduce these beliefs within both family and romantic contexts [24]. Contempt toward the mother and deferential treatment of the father may reflect internalized patriarchal values, where maternal authority is devalued and male authority figures are respected or feared. These dynamics could be associated with coercive behaviors in romantic relationships, especially when power and control are viewed as gendered and legitimate. Future research is encouraged to explore this phenomenon in more depth.

Adding to these profiles, lower levels of perpetrated cyber aggression were linked to the absence of variables explained above: experienced intimate partner violence, perpetrated intimate partner violence, psychopathy, and antisocial and law-violating behaviors, further supporting our established hypotheses. Due to variability issues, perpetrated cyber aggression could not be analyzed.

As per experienced cyber aggression, the pathways shown in fsQCA analyses are different across variables, but patterns can be identified. Multiple pathways were identified, reflecting the heterogeneous nature of victim profiles, which indicates that adolescents may become victims of CIPV through multiple trajectories. One important configuration included the absence of psychopathy and child-to-parent violence, indicating a low-risk profile—individuals who are not themselves aggressive but may be involved in asymmetric or coercive relationships. This could be explained by limited exposure to conflictual dynamics, fewer strategies for boundary-setting, or a tendency toward passive conflict management, as previous literature suggests [18]. Conversely, another equally relevant pathway featured the presence of psychopathy, antisocial behavior, and both perpetrated and experienced dating violence, in line with previous research [19,51]. This pattern likely reflects adolescents engaged in reciprocal cycles of aggression, where violence is normalized as part of their relational style [48,49]. These results could suggest a profile of mutual violence or “victim-perpetrators”, consistent with research showing bidirectional dynamics in adolescent relationships [5,31]. Perhaps the most consistent trait of the main profiles was the absence of child-to-parent violence towards both their mother and father, suggesting that they might be less experienced with conflictual dynamics, less likely to recognize early signs of coercion, or more inclined to adopt passive or compliant roles in romantic relationships.

Finally, regarding experienced cyber control, the analyses included various combinations of psychopathy, antisocial behavior, dating violence, and child-to-parent violence, suggesting again a bidirectional or mutually violent relationship structure. The overlap of both perpetrated and experienced dating violence in all configurations aligns with findings of perpetrated cyber control and reinforces the idea that control in relationships is often reciprocated or normalized, particularly when both partners show high-risk traits [11,13]. Results on lower experienced cyber control mirror these findings: the absence of antisocial behavior, dating violence, and child-to-parent violence emerges in the most relevant configurations, suggesting that adolescents embedded in non-violent relational environments and with low-risk individual profiles are significantly less likely to experience controlling behaviors from their partners, possibly due to healthier relationship models, greater assertiveness, and mutual respect within their romantic interactions. Altogether, these results highlight that both individual traits and relational histories play a key role in shaping adolescents’ vulnerability to cyber intimate partner violence. They support a view of CIPV not as a single-pathway phenomenon but as an outcome of diverse psychological and interpersonal dynamics. Specifically, these patterns highlight the protective nature of non-violent relational contexts and low-risk personality profiles, suggesting potential lines of intervention to prevent CIPV in Spanish adolescents.

Research and clinical implications

The present study offers important insights for the prevention and intervention of CIPV among adolescents. The results emphasize the need for professionals working with youth to assess not only romantic relationship dynamics but also online communication habits—particularly in contexts of conflict, control, or jealousy [29]. Family dynamics can also be a potential line of intervention, according to our findings, as child-to-parent violence is strongly associated with cyber aggression and control in romantic relationships in Spanish adolescents. The literature suggests that designing interventions based on attachment insecurity or parental styles could target the potential roots of behaviors and attitudes towards violence in children and adolescents, preventing maladaptive relationship dynamics from being transferred from the household to intimate relationships, especially in online settings [52]. These programs should also include gender-based perceptions of caregivers, as research consistently finds connections between aggressive or controlling behaviors in adolescents and their treatment of their parents [18,25]. Targeting underlying beliefs of sexism when addressing adolescents’ violent behaviors towards their mothers could prevent other forms of aggression in their intimate relationships, ultimately protecting both adolescents and female caregivers from these forms of violence [22,26,50].

These programs could also include high-risk individual factors that give adolescents a particular vulnerability towards CIPV. These could focus on teaching healthy communication skills and raising awareness of how individual traits like impulsivity or low empathy can shape dating behaviors. Since psychopathic traits, antisocial behavior, and previous experiences of violence (within the family or romantic contexts) emerged as key risk factors, interventions should target emotion regulation, empathy-building, and conflict resolution—especially in youth with behavioral or relational difficulties [13,20].

Importantly, interventions should also address harmful online practices, including exposure to websites or social media content that promote sexist beliefs, normalize violence in relationships, or reinforce toxic gender roles [4,30]. Adolescents frequently engage with online spaces that may encourage hostility, jealousy, and control under the guise of romantic interest—increasing the risk of CIPV [52]. The literature points at the strong connection between online and offline violence, suggesting that despite healthy family dynamics, adolescents are still exposed to harmful discourse about intimate and romantic relationships [28]. Thus, these findings could direct interventions to focus on debunking myths about digital control, teaching about ethical online practices, and encouraging adolescents to engage in positive communication with their intimate partners, ultimately preventing cyber dating violence perpetration and experience.

Limitations and future research

The present study is not without limitations. First, the study used a cross-sectional design, which limits the ability to make conclusions about cause and effect. Longitudinal studies would be useful to further understand associations between variables since cross-sectional data do not allow confirming the direction of these relationships. This kind of research could also explore how different types of violence appear and change throughout adolescence, helping us design better interventions adapted to specific life stages. Additionally, future studies could examine the role of other factors (e.g., attachment styles, trauma, or family dynamics) as potential mediators or moderators in these processes.

The second limitation addresses the characteristics of the sample. Convenience sampling was conducted; therefore, the results should be interpreted with caution. All participants came from educational settings, where the levels of dating violence and child-to-parent violence (CPV) tend to be moderate or low, potentially biasing results. More diversity is advised for future research to ensure generalizability; including cross-cultural young people from other environments, such as juvenile justice facilities, would ensure the representation of adolescents showing more serious or high-risk behaviors.

Third, we relied on self-reported questionnaires, introducing the possibility of bias. Although this method is useful for capturing adolescents’ personal experiences, it can be affected by social desirability or memory errors. Including reports from other sources, such as parents, teachers, or professionals, could help verify and enrich the data, offering a fuller view of the adolescents’ behavior and context.

Another limitation refers to the sample, as it was non-probabilistic, and the results should be interpreted with caution. Despite this, it is important to consider its potential representativeness in light of existing research on cyber victimization and perpetration among Spanish adolescents. While caution is warranted when generalizing the findings, the sample reflects key sociodemographic and behavioral trends commonly identified in Spanish youth populations, suggesting that the results may be informative for understanding the broader dynamics of cyber intimate partner violence in this context.

Finally, one notable limitation of the present study is the insufficient variability in the perpetrated cyber aggression subfactor, which prevented its inclusion in the fsQCA analyses. Several factors may account for this lack of variability. First, sample-related biases such as the relatively small sample size and the possibility of a restricted range in responses due to social desirability bias—especially given the sensitive nature of admitting to violent behaviors—may have led participants to underreport perpetration. In contrast, hierarchical regression models are able to accommodate limited variability and still produce interpretable results, whereas fsQCA requires sufficient distribution across conditions to identify meaningful configurational patterns. Furthermore, it is worth considering whether the instrument used to assess cyber aggression perpetration was sufficiently sensitive or whether alternative or complementary measures might capture a broader range of behaviors more effectively. Finally, this limitation could also reflect an inherent characteristic of the phenomenon itself, in which cyber aggression perpetration among adolescents may indeed occur less frequently or be less readily acknowledged, particularly in community-based samples. Future research should aim to replicate findings with larger and more diverse samples and explore the utility of other instruments or qualitative methods to capture the full spectrum of perpetrated behaviors in adolescent digital relationships.

Although our study examined both the perpetration and victimization of cyber intimate partner violence, future research could investigate the three distinct profiles that emerge in these settings: victims, perpetrators, and those who occupy both roles (dual-role individuals). Exploring these roles may help clarify whether CIPV reflects isolated victimization or is part of a broader pattern of mutual or escalating conflict within adolescent relationships. Furthermore, future research should consider including broader measures that encompass a wider range of online platforms and communication modalities to provide a more comprehensive understanding of adolescent cyberviolence.

Reflecting on these limitations could direct further research on this important topic, especially when designing intervention programs for adolescents with high-risk traits and behaviors who are more likely to engage in CIPV.

## 5. Conclusions

In conclusion, this study explored the predictors of cyber intimate partner violence in adolescents through both hierarchical regression models and fuzzy-set Qualitative Comparative Analysis. The findings highlight the complex interplay between individual traits—such as psychopathy and antisocial behavior—and relational experiences, including child-to-parent and dating violence, in shaping patterns of both perpetration and victimization in the digital sphere. The combination of linear and non-linear methodologies provided valuable nuance to the interpretation of our results, reaching conclusions that would have been unattainable by relying solely on linear analyses. The configurational approach provided by fsQCA analyses revealed that multiple risk pathways can lead to similar outcomes, emphasizing the need for flexible, tailored prevention actions.

These results underscore the importance of developing early intervention programs that address not only individual behavior but also family dynamics and the quality of adolescents’ romantic relationships. Schools, families, and community organizations must work together to foster empathy, emotional regulation, and healthy relationship skills among young people. By doing so, we can help create safer digital and offline spaces for adolescents—spaces where trust, respect, and connection take the place of control and harm. There is still much work to be done, but by listening to young people and acting early, we can help change their stories—for the better.

## Figures and Tables

**Table 1:** Mean comparison regarding gender.

Variable	Boys	Girls	Cohen’s *d*	*t*-Test	
	*M (SD)*	*M (SD)*		*t*	*p*
Psychopathy	4.46 (2.52)	3.72 (2.51)	0.30	2.02	0.020
A.behavior	6.59 (4.58)	4.16 (2.68)	0.72	3.28	0.001
CTPV mother	3.51 (3.69)	3.85 (2.96)	−0.10	−0.71	0.238
CTPV father	3.44 (4.06)	3.14 (2.82)	0.09	0.63	0.265
Perp. DV	7.41 (6.69)	7.94 (6.04)	−0.08	−0.56	0.287
Exp. DV	8.35 (7.75)	9.02 (8.47)	−0.08	−0.57	0.291
Perp. CIPV	0.15 (0.57)	0.09 (0.57)	0.11	0.77	0.221
Perp. CIPC	1.01 (1.95)	1.35 (2.00)	−0.17	−1.15	0.125
Exp. CIPV	0.21 (0.58)	0.15 (0.75)	0.09	0.62	0.268
Exp. CIPC	1.29 (1.99)	1.21 (1.87)	0.04	0.30	0.381

*Note: M:* mean; *SD*: standard deviation; β = standardized regression coefficient; *t*: Student’s *t* test statistic; *p*: probability. A.behavior = antisocial behavior; CTPV = child-to-parent violence; Perp. = perpetrated; Exp. = experienced; DV = dating violence; CIPV = cyber intimate partner violence; CIPC = cyber intimate partner control.

**Table 2:** Correlations between the dimensions of cyber intimate partner violence and its predictors.

	1	2	3	4	5	6	7	8	9	10
1. P	–									
2. AB	0.42 **	–								
3. CTPM	0.24 **	0.37 **	–							
4. CTPF	0.24 **	0.36 **	0.81 **	–						
5. PIPV	0.37 **	0.45 **	0.36 **	0.30 **	–					
6. EIPV	0.23 **	0.41 **	0.32 **	0.23 **	0.70 **	–				
7. PCIPV	0.14 *	0.26 **	0.17 *	0.05	0.30 **	0.32 **	–			
8. PCIPC	0.33 **	0.38 **	0.19 **	0.13	0.54 **	0.38 **	0.28 **	–		
9. ECIPV	0.11	0.28 **	0.19 **	0.04	0.32 **	0.50 **	0.66 **	0.20 **	–	
10. ECIPC	0.24 **	0.33 **	0.18 **	0.06	0.39 **	0.56 **	0.27 **	0.49 **	0.40 **	–

*Note*: P = psychopathy; CS = antisocial behaviors; CTPM = child-to-parent violence towards mother; CTPF = child-to-parent violence towards father; PIPV = perpetrated intimate partner violence; EIPV = experienced intimate partner violence; PCIPV = perpetrated cyber intimate partner violence; PCIPC = perpetrated cyber intimate partner control; ECIPV = experienced cyber intimate partner violence; ECIPC = experienced cyber intimate partner control; * *p* ≤ 0.01; ** *p* ≤ 0.001.

**Table 3:** Hierarchical regression model predicting cyber intimate partner violence.

		Criterion Variables															
		Perpetrated Cyber Intimate Partner Aggression				Perpetrated Cyber Intimate Partner Control				Experienced Cyber Intimate Partner Aggression				Experienced Cyber Intimate Partner Control			
Predictor		∆R^2^	∆F	*β*	VIF	∆R^2^	∆F	*β*	VIF	∆R^2^	∆F	*β*	VIF	∆R^2^	∆F	*β*	VIF
Step 1		0.08 **	50.53 **			0.23 ***	18.71 ***			0.08 **	5.67 **			0.15 ***	10.99 ***		
	Psychopathy			0.00	1.24			0.27 ***	1.24			0.06	1.23			0.21 *	1.24
	A.behavior			0.28 ***	1.24			0.30 ***	1.24			0.26 *	1.23			0.24 **	1.24
Step 2		0.08 **	5.84 **			0.02	1.24			0.07 **	5.28 **			0.03	2.28		
	Psychopathy			0.03	1.26			0.27 ***	1.26			0.08	1.25			0.23 **	1.26
	A.behavior			0.28 ***	1.41			0.27 ***	1.41			0.21 *	1.40			0.22 *	1.41
	CTPV mother			0.43 ***	2.98			0.21	2.98			0.47 ***	3.00			0.29 *	2.98
	CTPV father			−0.48 ***	2.96			−0.16	2.96			−0.39 **	2.97			−0.27	2.96
Step 3		0.11 ***	8.64 ***			0.18 ***	19.19 ***			0.22 ***	20.57 ***			0.21 ***	20.63 ***		
	Psychopathy			−0.01	1.35			0.19 *	1.35			0.03	1.33			0.22 **	1.35
	A.behavior			0.16	1.55			0.11	1.55			0.04	1.54			0.06	1.55
	CTPV mother			0.35 **	3.04			0.11	3.04			0.35 **	3.07			0.20	3.04
	CTPV father			−0.48 ***	2.97			−0.15	2.97			−0.38 ***	2.98			−0.28 *	2.97
	Perp. DV			0.15	2.30			0.39 ***	2.30			0.21 *	2.27			−0.02	2.30
	Exp. DV			0.25 *	2.11			0.15	2.11			0.37 ***	2.10			0.52 ***	2.11
Durbin–Watson		1.83				1.88				1.97				2.15			
R^2^ adj.		0.23 ***				0.40 ***				0.34 ***				0.36 ***			

*Note*: R^2^ adj = adjusted R^2^ value; ΔR^2^  = change in R^2^; ΔF = change in F; ß = regression coefficient; t  = t-value, VIF  = Variance Inflation Factor; A.behavior = antisocial behavior; CTPV = child-to-parent violence; Perp. DV = perpetrated dating violence; Exp. DV = experienced dating violence; * *p* ≤ 0.05; ** *p* ≤ 0.01; *** *p* ≤ 0.001.

**Table 4:** Main descriptions and calibration values.

	Psychopathy	Antisocial Behavior	CTP Violence Towards Mother	CTP Violence Towards Father	Perpetrated Intimate Partner Violence	Experienced Intimate Partner Violence	Perpetrated Cyber Aggression	Perpetrated Cyber Control	Cyber Victimization	Experienced Cyber Control
*M*	4.00	4.92	3.71	3.19	8.14	9.13	0.10	1.30	0.16	1.28
*SD*	2.56	3.56	3.17	3.23	6.48	8.28	0.55	2.02	0.67	1.96
Min.	0	0	0	0	0	0	0	0	0	0
Max	13.00	22.00	20	23.00	39.00	53.00	6.00	12.00	7.00	11.00
P10	1.00	1.00	1.00	0	1.00	1.00	0	0	0	0
P50	4.00	4.00	3.00	3.00	6.00	7.00	0	1.00	0	1.00
P90	7.30	9.90	8.00	7.00	16.00	19.40	0	3.00	0	4.00

*Note*: M = mean; SD = standard deviation; Min. = minimum; Max. = maximum; P10 = 10th percentile; P50 = 50th percentile. CTP = child-to-parent. P90 = 90th percentile. Higher calibration values indicate a stronger presence of the condition.

**Table 5:** Necessity analyses for cyber intimate partner violence.

	High Levels of Cyber Control Perpetrated		Low Levels of Cyber Control Perpetrated		High Levels of Cyber Victimization		Low Levels of Cyber Victimization		High Levels of Cyber Control Received		Low Levels of Cyber Control Received	
	Cons.	Cov.	Cons.	Cov.	Cons.	Cov.	Cons.	Cov.	Cons.	Cov.	Cons.	Cov.
Psychopathy	0.78	0.60	0.78	0.60	0.76	0.72	0.73	0.62	0.80	0.57	0.57	0.62
No Psychopathy	0.46	0.44	0.46	0.44	0.60	0.72	0.67	0.71	0.47	0.41	0.61	0.82
Antisocial Behavior	0.72	0.63	0.72	0.63	0.69	0.75	0.68	0.65	0.74	0.60	0.51	0.64
No Antisocial Behavior	0.53	0.44	0.53	0.44	0.68	0.71	0.74	0.68	0.56	0.42	0.68	0.80
CTP mother	0.69	0.61	0.69	0.61	0.67	0.75	0.66	0.65	0.71	0.58	0.50	0.63
No CTP mother	0.55	0.45	0.55	0.45	0.68	0.70	0.74	0.67	0.55	0.41	0.67	0.78
CTP father	0.63	0.59	0.63	0.59	0.63	0.74	0.63	0.65	0.66	0.57	0.50	0.67
No CTP father	0.61	0.47	0.61	0.47	0.63	0.74	0.74	0.64	0.61	0.44	0.68	0.76
Perpetrated PV	0.79	0.69	0.79	0.69	0.71	0.77	0.68	0.65	0.76	0.61	0.49	0.61
No Perpetrated PV	0.47	0.40	0.47	0.40	0.67	0.71	0.75	0.70	0.51	0.39	0.68	0.81
Experienced PV	0.79	0.70	0.79	0.70	0.72	0.80	0.69	0.67	0.80	0.65	0.46	0.58
No Experienced PV	0.49	0.40	0.49	0.40	0.70	0.72	0.79	0.72	0.49	0.37	0.73	0.85

*Note*: Cons.: consistency; Cov.: coverage; condition needed: consistency ≥ 0.90. CTP = child-to-parent; PV = partner violence.

**Table 6:** Sufficiency analyses for predictors of cyber intimate partner violence.

Frequency Cutoff: 1	Perpetrated Cyber Control			~Perpetrated Cyber Control			Cyber Victimization			~Cyber Victimization			Experienced Cyber Control			~Experienced Cyber Control		
	Consistency Cutoff: 0.81			Consistency Cutoff: 0.81			Consistency Cutoff: 0.89			Consistency Cutoff: 0.95			Consistency Cutoff: 0.81			Consistency Cutoff: 0.90		
	1	2	3	1	2	3	1	2	3	1	2	3	1	2	3	1	2	3
Psychopathy	●		●			○	○		●			●	●	●				
Antisocial behavior			●			○		○	●		○	○	●	●	○	○	○	
CTP violence towards mother		●					○	○				○		○	●		○	○
CTP violence towards father			○				○	○			●		○		●			○
Perpetrated PV	●	●			○				●	●			●	●	●	○		○
Experienced PV	●	●	●	○				○	●	○	○	○	●	●	●	○	○	○
Raw coverage	0.62	0.54	0.35	0.75	0.73	0.51	0.46	0.44	0.46	0.49	0.38	0.36	0.35	0.33	0.31	0.49	0.46	0.44
Unique coverage	0.05	0.02	0.01	0.04	0.03	0.01	0.02	0.01	0.02	0.05	0.02	0.03	0.04	0.02	0.13	0.06	0.02	0.03
Consistency	0.80	0.80	0.83	0.83	0.83	0.84	0.81	0.85	0.90	0.93	0.92	0.94	0.81	0.80	0.83	0.92	0.94	0.91
Overall solutions coverage	0.72			0.87			0.96			0.77			0.51			0.72		
Overall solutions consistency	0.77			0.77			0.74			0.88			0.81			0.88		

*Note:* ~: absence of condition; ● = presence of condition, ○ = absence of condition. Expected vector for perpetrated cyber control (1, 1, 1, 1, 1, 0), for ~perpetrated cyber control (0, 0, 0, 0, 0, 1), for cyber victimization (0, 0, 0, 0, 0, 1), for ~cyber victimization (1, 1, 1, 1, 1, 0), for experienced cyber control (0, 0, 0, 0, 0, 1) and for ~experienced cyber control (1, 1, 1, 1, 1, 0) using the format of 47. CTP = child-to-parent; PV = partner violence.

## Data Availability

Data from the study can be made available upon reasonable request to the corresponding author due to ethical considerations and the sensitive nature of the information collected.

## References

[1] Garaigordobil M., Martínez-Valderrey V. (2018). Technological Resources to Prevent Cyberbullying During Adolescence: The Cyberprogram 2.0 Program and the Cooperative Cybereduca 2.0 Videogame. Front. Psychol..

[2] Zhang D., Huebner E.S., Tian L. (2020). Longitudinal Associations among Neuroticism, Depression, and Cyberbullying in Early Adolescents. Comput. Hum. Behav..

[3] Lamash L., Fogel Y., Hen-Herbst L. (2024). Adolescents’ Social Interaction Skills on Social Media versus in Person and the Correlations to Well-Being. J. Adolesc..

[4] Afrouz R., Vassos S. (2024). Adolescents’ Experiences of Cyber-Dating Abuse and the Pattern of Abuse Through Technology, A Scoping Review. Trauma Violence Abus..

[5] Gilbar O., Charak R., Trujillo O., Cantu J.I., Cavazos V., Lavi I. (2023). Meta-Analysis of Cyber Intimate Partner Violence Perpetration and Victimization: Different Types and Their Associations with Face-to-Face IPV among Men and Women. Trauma Violence Abus..

[6] Cava M.-J., Buelga S., Cava M.-J., Buelga S. (2018). Propiedades Psicométricas de La Escala de Ciber-Violencia En Parejas Adolescentes (Cib-VPA). Suma Psicol..

[7] Redondo I., Ozamiz-Etxebarria N., Jaureguizar J., Dosil-Santamaria M. (2024). Cyber Dating Violence: How Is It Perceived in Early Adolescence?. Behav. Sci..

[8] Machado B., de Faria P.L., Araújo I., Caridade S. (2024). Cyber Interpersonal Violence: Adolescent Perspectives and Digital Practices. Int. J. Environ. Res. Public Health.

[9] Salmela-Aro K. (2011). Stages of Adolescence. Encycl. Adolesc..

[10] Liu S., Xu B., Zhang D., Tian Y., Wu X. (2022). Core Symptoms and Symptom Relationships of Problematic Internet Use Across Early, Middle, and Late Adolescence: A Network Analysis. Comput. Hum. Behav..

[11] Torp Løkkeberg S., Ihlebæk C., Brottveit G., Del Busso L. (2024). Digital Violence and Abuse: A Scoping Review of Adverse Experiences Within Adolescent Intimate Partner Relationships. Trauma Violence Abus..

[12] Cheng T.W., Mills K.L., Pfeifer J.H. (2024). Revisiting Adolescence as a Sensitive Period for Sociocultural Processing. Neurosci. Biobehav. Rev..

[13] Alsoubai A., Razi A., Agha Z., Ali S., Stringhini G., Chodhury M.D.E., Wisniewski P.J. (2024). Profiling the Offline and Online Risk Experiences of Youth to Develop Targeted Interventions for Online Safety. Proc. ACM Hum. Comput. Interact..

[14] Matud M.P. (2024). Gender Differences in Sexting and Its Association with Well-Being and Intimate Partner Violence Victimization from Adolescence to Old Age. Sexes.

[15] Longobardi C., Fabris M.A., Prino L.E., Settanni M. (2021). Online Sexual Victimization among Middle School Students: Prevalence and Association with Online Risk Behaviors. Int. J. Dev. Sci..

[16] Pineda D., Galán M., Martínez-Martínez A., Campagne D.M., Piqueras J.A. (2022). Same Personality, New Ways to Abuse: How Dark Tetrad Personalities Are Connected with Cyber Intimate Partner Violence. J. Interpers. Violence.

[17] De Brito S.A., Forth A.E., Baskin-Sommers A.R., Brazil I.A., Kimonis E.R., Pardini D., Frick P.J., Blair R.J.R., Viding E. (2021). Psychopathy. Nat. Rev. Dis. Primers.

[18] Calvete E., Orue I., Gámez-Guadix M. (2013). Child-to-Parent Violence: Emotional and Behavioral Predictors. J. Interpers. Violence.

[19] Frick P.J., Marsee M.A., Patrick C. (2005). Psychopathy and Developmental Pathways to Antisocial Behavior in Youth. Handbook of Psychopathy.

[20] Shaffer C.S., Gatner D.T., McCuish E., Douglas K.S., Viljoen J.L. (2021). The Role of Psychopathic Features and Developmental Risk Factors in Trajectories of Physical Intimate Partner Violence. Psychol. Violence.

[21] Espuig A., Lacomba-Trejo L., González-Sala F. (2025). Child-to-Parent Violence Among Adolescents: A Preliminary Analysis of Its Association with Sociodemographic Variables, Dating Violence, and Antisocial Traits. Children.

[22] Erostarbe I.I., Arnoso Martínez A., Astondoa E.E. (2018). Prominent Intervention Programs in Child-to-Parent Violence. Papeles Del Psicól./Psychol. Pap..

[23] Cano-Lozano M.C., León S.P., Contreras L. (2021). Child-to-Parent Violence: Examining the Frequency and Reasons in Spanish Youth. Fam. Relat..

[24] Junco-Guerrero M., Fernández-Baena F.J., Cantón-Cortés D. (2025). Risk Factors for Child-to-Parent Violence: A Scoping Review. J. Fam. Violence.

[25] Calvete E., Orue I., Fernández-González L., Chang R., Little T.D. (2020). Longitudinal Trajectories of Child-to-Parent Violence through Adolescence. J. Fam. Violence.

[26] Rogers M.M., Ashworth C. (2024). Child-to-Parent Violence and Abuse: A Scoping Review. Trauma Violence Abus..

[27] Fishbein M., Ajzen I. (2011). Predicting and Changing Behavior: The Reasoned Action Approach.

[28] Donato S., Eslen-Ziya H., Mangone E. (2022). From Offline to Online Violence: New Challenges for the Contemporary Society. Int. Rev. Sociol..

[29] Núñez A., Álvarez-García D., Pérez-Fuentes M.-C. (2021). Anxiety and Self-Esteem in Cyber-Victimization Profiles of Adolescents. Comun. Media Educ. Res. J..

[30] Álvarez-García D., Núñez Pérez J.C., Dobarro González A., Rodríguez Pérez C. (2015). Risk Factors Associated with Cybervictimization in Adolescence. Int. J. Clin. Health Psychol..

[31] Fernet M., Lapierre A., Hébert M., Cousineau M.M. (2019). A Systematic Review of Literature on Cyber Intimate Partner Victimization in Adolescent Girls and Women. Comput. Hum. Behav..

[32] Virós-Martín C., Jiménez-Morales M., Montaña-Blasco M. (2025). Adolescentes, TikTok e Instagram: Percepciones Sobre El Impacto de Las Tecnologías Digitales En Su Vida Social. Rev. De Comun..

[33] Iñigo A., Fernández L., Tomasena J.M. (2024). Disinterest, Normalisation of Gender Violence and Fear of Being Cancelled: Mediatised Learning on Antifeminist and Anti-LGBTIQ+ Discourses among Teenagers in Barcelona. Int. Commun. Gaz..

[34] Benítez-Hidalgo V., Henares-Montiel J., Ruiz-Pérez I., Pastor-Moreno G. (2024). International Prevalence of Technology-Facilitated Sexual Violence Against Women: A Systematic Review and Meta-Analysis of Observational Studies. Trauma Violence Abus..

[35] Soriano-Ayala E., Cala V.C., Orpinas P. (2023). Prevalence and Predictors of Perpetration of Cyberviolence Against a Dating Partner: A Cross-Cultural Study with Moroccan and Spanish Youth. J. Interpers. Violence.

[36] Salekin R.T., Ziegler T.A., Larrea M.A., Anthony V.L., Bennett A.D. (2003). Predicting Dangerousness with Two Millon Adolescent Clinical Inventory Psychopathy Scales: The Importance of Egocentric and Callous Traits. J. Pers. Assess..

[37] Cooke D.J., Michie C., Skeem J. (2007). Understanding the Structure of the Psychopathy Checklist–Revised: An Exploration of Methodological Confusion. Br. J. Psychiatry.

[38] Andreu J.M., Peña M.E. (2013). Propiedades Psicométricas de La Escala de Conducta Antisocial y Delictiva En Adolescentes. An. De Psicol..

[39] Penado M., Andreu J.M., Peña E. (2014). Agresividad Reactiva, Proactiva y Mixta: Análisis de Los Factores de Riesgo Individual. Anu. Psicol. Juríd..

[40] Gámez-Guadix M., Straus M.A., Carrobles A., Muñoz-Rivas M.J., Almendros C. (2010). Corporal Punishment and Long-Term Behavior Problems: The Moderating Role of Positive Parenting and Psychological Aggression. Psicothema.

[41] Straus M.A., Douglas E.M. (2004). A Short Form of the Revised Conflict Tactics Scales, and Typologies for Severity and Mutuality. Violence Vict..

[42] Grupo Lisis Conflict Tactics Scales (CTS2). https://lisis.blogs.uv.es/instrumentos-2013-2016/.

[43] Fernández-Fuertes A.A., Fuertes A., Pulido R.F. (2006). Evaluación de La Violencia En Las Relaciones de Pareja de Los Adolescentes. Validación Del Conflict in Adolescent Dating Relationships Inventory (CADRI)-Versión Española 1. Int. J. Clin. Health Psychol..

[44] Wolfe D.A., Scott K., Reitzel-Jaffe D., Wekerle C., Grasley C., Straatman A.L. (2001). Development and Validation of the Conflict in Adolescent Dating Relationships Inventory. Psychol. Assess..

[45] Ragin C.C. (2008). Redesigning Social Inquiry. Fuzzy Sets and Beyond.

[46] Eng S., Woodside A.G. (2012). Configural Analysis of the Drinking Man: Fuzzy-Set Qualitative Comparative Analyses. Addictive Behaviors.

[47] Pappas I.O., Woodside A.G. (2021). Fuzzy-set Qualitative Comparative Analysis (fsQCA): Guidelines for research practice in Information Systems and marketing. Int. J. Inf. Manag..

[48] Giroux S., Guay M.C. (2022). Assessing the Contribution of Callous–Unemotional Traits and Affective Empathy to Aggressive Behaviour Among Teenagers Hosted in a Youth Protection Centre. Psychol. Crime Law.

[49] Díaz-Galván K.X., Ostrosky-Shejet F., Romero-Rebollar C. (2015). Cognitive and Affective Empathy: The Role in Violent Behavior and Psychopathy. Rev. Médica Del. Hosp. General. México.

[50] Loinaz I., Barboni L., Ma-de-Sousa A. (2020). Gender Differences in Child-to-Parent Violence Risk Factors. An. Psicol..

[51] Damra J.K., Abujilban S., Akour M.M. (2024). The Cyber Intimate Partner Violence: Prevalence, Context, and Relationship With In-Person Intimate Violence Victimization. J. Fam. Issues.

[52] Laforte S., Paradis A., Todorov E.H., Cyr C. (2023). Romantic Attachment and Cyber Dating Violence in Adolescence: A Dyadic Approach. J. Adolesc..

